# Antifibrosis Efficacy of Apo-9-Fucoxanthinone-Contained *Sargassum horneri* Ethanol Extract on Nasal Polyp: An In Vitro and Ex Vivo Organ Culture Assay

**DOI:** 10.3390/cimb44110395

**Published:** 2022-11-21

**Authors:** Mi-Jin Yim, Jeong Min Lee, Seok-Chun Ko, Hyun-Soo Kim, Ji-Yul Kim, Seong Kook Park, Dae-Sung Lee, Il-Whan Choi

**Affiliations:** 1 National Marine Biodiversity Institute of Korea, Seocheon 33662, Republic of Korea; 2 Department of Otorhinolaryngology-Head & Neck Surgery, Busan Paik Hospital, Inje University College of Medicine, Busan 47392, Republic of Korea; 3 Department of Microbiology and Immunology, College of Medicine Inje University, Busan 47392, Republic of Korea

**Keywords:** nasal polyps, *Sargassum horneri*, Apo-9-fucoxanthinone, extracellular matrix, α-SMA, air-liquid interface organ culture

## Abstract

*Sargassum horneri* is a seaweed species with diverse bioactivities. However, its antifibrotic effects during nasal polyp (NP) formation are not clearly understood. Therefore, we investigated the inhibitory effect of *S. horneri* on fibrosis progression in NP-derived fibroblasts (NPDFs) and NP tissues ex vivo. NPDFs were stimulated with TGF-β1 in the presence or absence of *S. horneri* ethanol extract (SHE). The extracellular matrix (ECM) protein production levels, myofibroblast differentiation (α-smooth muscle actin, α-SMA), and phosphorylation of Smad 2/3 and -ERK in TGF-β1-stimulated NPDFs were investigated using western blotting. Further, the contractile activity of SHE was assessed by performing a collagen gel contraction assay. The expression levels of collagen-1, fibronectin, and α-SMA were investigated in NP organ cultures treated with SHE. TGF-β1 stimulated ECM protein expression, myofibroblast differentiation, and collagen contractile activity while these were attenuated by pretreatment with SHE. We also found antifibrotic effect of SHE on ex vivo NP tissues. The antifibrotic effects of SHE were modulated through the attenuation of Smad 2/3 and ERK signaling pathways in TGF-β1-stimulated NPDFs. In conclusion, SHE inhibited ECM protein accumulation and myofibroblast differentiation during NP remodeling. Thus, SHE may be helpful as a treatment for NP recurrence after endoscopic sinus surgery.

## 1. Introduction

Nasal polyp (NP) is a chronic inflammatory disease of the upper airway, nose, and sinuses, in which abnormal protruding tissue swellings of the nasal mucosa appear [1]. NPs are estimated to occur in 1–4% of the general population. NPs derived from the middle meatus are soft, pinkish, teardrop-shaped noncancerous outgrowths inside the nasal cavities that exhibit symptoms such as rhinorrhea, hyposmia, nasal congestion, and headaches [2,3]. NPs are typically characterized by abnormal tissue remodeling, focal fibrosis, edema, pseudocyst formation, basement membrane thickening, epithelial layer proliferation, goblet cell hyperplasia, and inflammatory cell infiltration of the stromal layer [4,5,6]. Because NPs usually recur after endoscopic sinus surgery, many patients require additional management. However, the causes and pathophysiology of NP formation are not clearly understood. The formation of NPs is thought to proceed through fibroblast differentiation into myofibroblasts and extracellular matrix (ECM) protein accumulation, commonly known as a hallmark of the fibrotic response [7].

Many cell types are involved in the pathogenesis of NPs, including goblet cells, eosinophils, mast cells, lymphocytes, and fibroblasts [8]. Among the many cells present in NPs, fibroblasts are the primary cell type of the NP architecture and contribute to NP remodeling [9]. Fibroblasts are abundant in the stroma of NPs, which are NP-derived fibroblasts (NPDFs). Fibroblasts, usually large flat spindle-shaped cells, are the most common connective tissue cells found in animal bodies [10]. Fibroblasts play a biological role in wound healing, angiogenesis, and inflammation. Fibroblasts are the central effector cells in fibrosis. Following activation by profibrotic stimuli, fibroblasts differentiate into myofibroblasts, leading to excessive ECM protein production, such as collagen and fibronectin [11]. Fibronectin is a multifunctional molecular glycoprotein involved in tissue remodeling. α-smooth muscle actin (α-SMA) expression is the most reliable marker of myofibroblastic phenotype differentiation.

Marine algae have traditionally been used as a food and medicine in Asian countries, including Korea, Japan, and China. Marine algae contain essential nutrients, such as dietary fibers, essential amino acids, minerals, vitamins, proteins, and polyunsaturated fatty acids [12]. Furthermore, marine algae have a wide range of biological activities, including antioxidant, anti-inflammatory, immunomodulatory, antiallergic, antibacterial, antiviral, anticancer, and anticoagulant effects [13,14]. In addition, marine algae contain many bioactive compounds such as proteins, minerals, sterols, polyunsaturated fatty acids, terpenoids, and amines [15]. Therefore, marine algae are fascinating sources of human nutrition and pharmaceuticals. *Sargassum horneri* (*S. horneri*) is a rich source of nutrients such as dietary fibers, vitamins, amino acids, and polysaccharides [16]. *S. horneri* has diverse bioactivities, including antiosteoporosis, antioxidant, anticoagulant, antiallergic, anticancer, and antiviral effects [17,18,19,20,21,22,23]. However, the antifibrotic activity and regulatory mechanism of *S. horneri* in NPDFs have not yet been reported.

In Korea, many drifting *S.horneri* occur, causing many problems, such as damage to the farms, odors, and landscape damage to the beach [24]. Rarely consumed as food in Korea, most of them are discarded. Therefore, we conducted this study to further increase the use value of *S. horneri*. We evaluated the effect of *S. horneri* ethanol extracts (SHE) on TGF-β1-induced ECM expression and myofibroblast differentiation in NPDFs and examined its regulatory mechanism. In addition, we investigated the antifibrotic effect of SHE in the organ culture of NPs that mimics the conditions for NP formation to examine their efficacy in humans.

## 2. Materials and Methods

### 2.1. Reagents

SHE was kindly provided by Dr. M. -J. Yim (National Marine Biodiversity Institute of Korea, Seocheon, Korea). We purchased TGF-β1 from R&D Systems (Minneapolis, MN, USA). SIS3 (cat. No. S0447) was purchased from Sigma-Aldrich (St. Louis, MO, USA). Antibodies against p-Smad 2 (cat. no. 3101) and p-Smad 3 (cat. no. 9520) were purchased from Cell Signaling Technology Inc. (Danvers, MA, USA). Antibodies against fibronectin (FN; cat. no. 610077) were purchased from BD Biosciences (San Jose, CA, USA). Rat tail type 1 collagen (cat. no. 354236) was purchased from BD Biosciences (San Jose, CA, USA). Antibodies against α-SMA (cat. no. ab5694) and collagen-1 (cat. no. ab88147) were purchased from Abcam, Inc. (Cambridge, MA, USA). Antibodies against goat anti-mouse IgG-HRP conjugate (cat. No. LF-SA8001) and GAPDH (cat. No. LF-PA0018) were purchased from Young In Frontier (Seoul, Korea). Antibodies against phospho (p)-extracellular signal-related kinase (ERK) (cat. no. 9106), c-Jun N-terminal kinase (JNK) (cat. no. 9252), p-JNK (cat. no. 9251), p-p38 mitogen-activated protein kinase (MAPK) (cat. no. 9211), Akt (cat. no. 9272), and p-Akt (cat. no. 4058) were purchased from Cell Signaling Technology, Inc. (Danvers, MA, USA). Antibodies against ERK (cat. no. sc-94), and p38 MAPK (cat. no. sc-535) were purchased from Santa Cruz Biotechnology, Inc. (Santa Cruz, CA, USA). Cell Counting Kit-8 (CCK-8) was purchased from Dojindo Laboratories (Kumamoto, Japan).

### 2.2. Preparation of SHE

*S. horneri* was harvested in Yangyang, Korea. SHE was prepared as previously reported by Kim et al. [25]. Briefly, *S. horneri* was washed with tap water and stored at −20 °C. The frozen samples were lyophilized and homogenized using a grinder. The dried powder was extracted with 70% (*v*/*v*) EtOH (1:10 *w*/*v*) for 1 h (five repeats) by sonication, and the extract was evaporated to dryness in vacuo. The powder was dissolved in 70% (*v*/*v*) EtOH before use in experiments for bioactivity assay.

### 2.3. NPDF Culture and Cell Viability Assay

Patients with NPs were recruited and NPDFs were cultured as previously reported [26]. This study was approved by the local ethics committee of Inje University, Busan Paik Hospital, Busan, Republic of Korea (Approval code: 80/2020). The purity of NPDFs was verified using a fibroblast marker antibody panel (cat. No. ab254015, Abcam Inc., Cambridge, MA, USA) and the characteristic cell morphology. NPDFs were used in the fourth to sixth cell passage. Cell viability was evaluated using the CCK-8 assay. Briefly, NPDFs (1 × 10^5^ cells/well) were cultured in a 96-well microplate in Dulbecco’s Modified Eagle Medium (DMEM). The NPDFs were treated with various SHE concentrations (30, 50, and 100 μg/mL). After incubation for 24 h at 37 °C (5% humidified CO_2_), an absorbance analysis at 450 nm was performed using a microplate reader (SpectraMax M2e, Molecular Devices, Sunnyvale, CA, USA). All assays were conducted in triplicates.

### 2.4. Western Blotting

NPDF lysates were collected in lysis buffer (G-Biosciences, St. Louis, MO, USA) with a protease inhibitor cocktail (Roche Diagnostics, Mannheim, Germany). Equal amounts of protein from cell lysates were separated using 10% sodium dodecyl sulfate-polyacrylamide mini-gel electrophoresis and transferred onto nitrocellulose membranes (GE Healthcare-Life Sciences, Chalfont, UK). Following overnight incubation with the specific primary antibody (α-SMA, Col-1, fibronectin, p-Smad2 and p-Smad3), membranes were incubated with a secondary antibody (goat anti-mouse IgG) conjugated to horseradish peroxidase. Immunoreactive bands were visualized using an enhanced chemiluminescence detection system (Pierce Biotechnology, Inc. Rockford, IL, USA). Band images were captured and analyzed using an imaging system (AI 600, GE Healthcare Life Sciences, Canton, MA, USA) and ImageJ software (ver. 1.52a; National Institutes of Health, Bethesda, MD, USA).

### 2.5. Preparation of Nuclear Extracts and EMSA

Nuclear extracts were prepared using the NE-PER^TM^ Nuclear Extraction Reagent (Pierce Biotechnology, Inc.). An oligonucleotide containing the immunoglobulin κ-chain binding site (κB, 5′-GATCTCAGA GGGGACTTTCCGAGAGA-3′) was synthesized as a probe for the DNA-protein binding assay. The 3′ end of the probe was labeled with biotin (Pierce Biotechnology, Inc.). Briefly, the binding reactions contained 5 mg of nuclear protein extract and 20 fM of biotin-labeled DNA. The reaction mixtures were incubated for 20 min at 27 °C in a final volume of 20 μL. Competition reactions were conducted by adding a 100-fold excess of unlabeled p65 NF-κB to the reaction mixture. The mixture was separated by electrophoresis and transferred to nylon membranes. Biotin-labeled DNA was detected using a LightShift chemiluminescent EMSA kit (Pierce Biotechnology, Inc.).

### 2.6. Collagen Gel Contraction Assay

The ability of the NPDFs to contract collagen gels was evaluated as previously described [27]. Briefly, rat tail type I collagen was diluted with serum-free DMEM to a concentration of 1 mg/mL and mixed with 1 N NaOH. NPDFs were then added to a 24-well cell culture plate to reach a final concentration of 1 × 10^5^ cells/mL in 500 μL. After the cell–collagen mixtures were allowed to solidify at 37 °C for 30 min, the plates were treated with SHE and TGF-β1 at the indicated concentrations. The plates were then incubated for 3 days. Finally, the gel sizes were measured using the ImageJ software.

### 2.7. Organ Culture of NPs

NP tissues were cultured using the air–liquid interface organ culture method. Patients with NPs were recruited as previously reported by Park et al. [28]. This study was approved by the local ethics committee of Inje University, Busan Paik Hospital, Busan, Republic of Korea. NPs were obtained from the region of the middle meatus and cut into small pieces (3 mm^3^). To investigate the inhibitory effects of SHE on NP formation, tissue fragments were saturated for 1 h in DMEM in the presence or absence of SHE (100 μg/mL). The tissue fragments were then placed on hydrated 1 × 1 cm gelatin sponges (Spongostan; Johnson & Johnson, Austin, TX, USA), with the mucosae facing upward and the submucosae facing downward. The gelatin sponge on which the NP tissue was placed was inserted into the wells of a 6-well plate containing 3 mL of the culture solution. The plates were placed in a 5% humidified CO_2_ incubator for 24 h.

### 2.8. Statistical Analysis

Data are presented as mean ± standard error of the mean. All statistical analyses were performed using GraphPad Prism software (ver. 5.0; GraphPad Software Inc., La Jolla, CA, USA). Dunnett’s multiple range test was used for comparisons between the groups.

## 3. Results

### 3.1. Effects of SHE on the Viability of NPDFs

**Figure 1:**
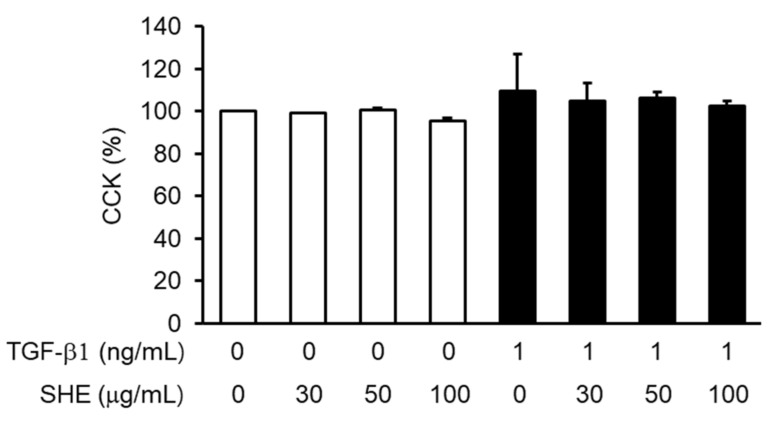
The effect of *Sargassum horneri* ethanol extracts (SHE) on nasal polyp–derived fibroblasts (NPDF) viability. The cells were treated with various concentrations (30, 50, and 100 μg/mL) of SHE for 24 h. Cell viability was assessed using the Cell Counting Kit-8 (CCK-8) assay, and the results are expressed as the percentage of surviving cells relative to the untreated cells. Each value indicates the mean ± standard error of the mean (SEM) and is representative of results obtained from three independent experiments.

NPDFs were treated with SHE at 30–100 μg/mL concentration. There was no cytotoxicity to NPDFs at SHE doses up to 100 μg/mL (Figure 1). Based on these results, a follow-up experiment was conducted with non-toxic concentrations of SHE (30–100 μg/mL).

### 3.2. Effect of SHE on Fibronectin, Collagen-1, and α-SMA Protein Expression in TGF-β1-Stimulated NPDFs

**Figure 2:**
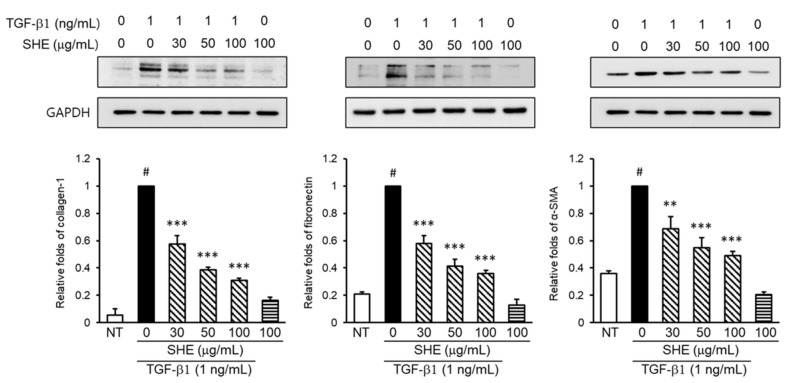
Effect of SHE on collagen-1, fibronectin, and α-smooth muscle actin (α-SMA) protein expression by TGF-β1-stimulated NPDFs. The cells were seeded at 6 × 10^4^ cells/mL and incubated with various concentrations (30, 50, and 100 μg/mL) of SHE for 1 h prior to TGF-β1 stimulation (1 ng/mL). Following stimulation with TGF-β1 for 24 h, collagen-1, fibronectin, and α-SMA protein expressions were determined by western blotting. GAPDH was used as an internal control. Each value indicates the mean ± SEM (*n* = 3) and is representative of results obtained from three independent experiments. # *p* < 0.05 vs. control group (no treatment, NT); ** *p* < 0.01 and *** *p* < 0.001 vs. TGF-β1-stimulated group.

First, we investigated the antifibrotic efficacy of SHE. We assessed the expression levels of profibrotic mediators such as collagen-1, fibronectin, and α-SMA in TGF-β1-induced NPDFs. Various concentrations of SHE (30–100 μg/mL) were administered to cells for 1 h before TGF-β1 stimulation for 24 h. In this study, TGF-β1 significantly increased the levels of profibrotic mediators in NPDFs. However, pretreatment with SHE significantly attenuated the expression levels of collagen-1, fibronectin, and α-SMA in TGF-β1-stimulated NPDFs (Figure 2).

### 3.3. SHE Inhibits TGF-β1-Stimulated Smad 2/3-Dependent Signaling Pathways

**Figure 3:**
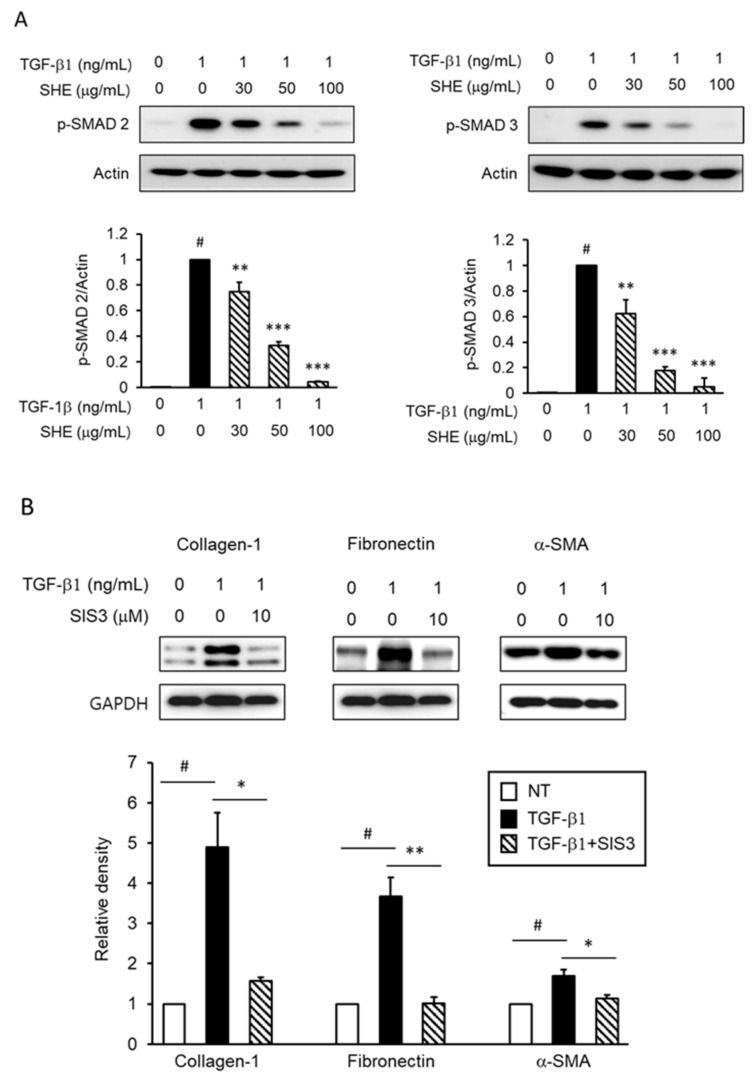
Effect of SHE on Smad 2/3 phosphorylation in TGF-β1-induced NPDFs. (**A**) The cells were treated with the indicated SHE concentrations (30, 50, and 100 μg/mL) for 1 h before stimulation with TGF-β1 (1 ng/mL) for 1 h. Nuclear protein extracts were then prepared and subjected to western blotting with antibodies specific for the phosphorylated forms of Smad 2 and Smad 3. The results presented are representative of three independent experiments; (**B**) The NPDFs were treated with 10 mM of Smad 3 specific inhibitor (SIS3) for 1 h, prior to stimulation with TGF-β1 (1 ng/mL) for 24 h. Each bar represents the mean ± SEM (*n* = 3) from three independent experiments. # *p* < 0.05 vs. control group (no treatment group); * *p* < 0.05, ** *p* < 0.01, and *** *p* < 0.001 vs. TGF-β1-stimulated group.

To understand the signaling pathways involved in TGF-β1-induced expression of profibrotic factors, we investigated canonical and non-canonical signal responses. Smad pathways, which are canonical signaling pathways, are a critical mechanism of signals from the TGF-β1 receptor; therefore, we assessed the effect of SHE on the phosphorylation of Smad 2/3 (p-Smad 2/3). p-Smad 2/3 was significantly enhanced in NPDFs stimulated with TGF-β1. However, when NPDFs were treated with SHE (30–100 μg/mL) for 1 h before TGF-β1 stimulation for 1 h, the expression level of p-Smad 2/3 was significantly suppressed (Figure 3A). To demonstrate whether Smad 2/3 pathways are critical for the augmented expression of collagen-1, fibronectin, and α-SMA with TGF-β1-stimulation in NPDFs, we treated NPDFs with a selective inhibitor of Smad 3 (SIS3, 10 μM). The expression levels of collagen-1, fibronectin, and α-SMA were assessed using western blotting. As expected, SIS3 significantly attenuated ECM expression and myofibroblast differentiation in TGF-β1-stimulated NPDFs (Figure 3B).

### 3.4. SHE Inhibits TGF-β1-Stimulated Smad 2/3-Independent Signaling Pathways

**Figure 4:**
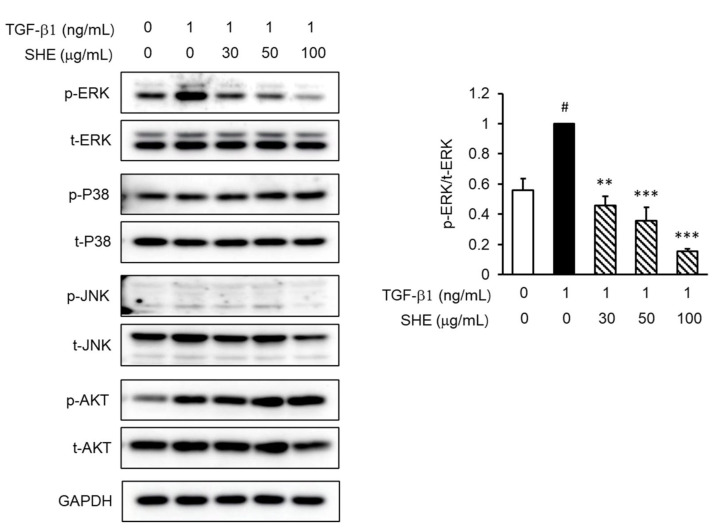
Effect of SHE on MAPK and PI3K/Akt phosphorylation in TGF-β1-stimulated NPDFs. The cells were treated with the indicated SHE concentrations (30, 50, and 100 μg/mL) for 1 h before stimulation with TGF-β1 (1 ng/mL) for 15 min. Western blotting was then performed with antibodies specific for the phosphorylated form of MAPK and PI3K/Akt with cytosol protein extract. The results presented are representative of three independent experiments. Each bar represents the mean ± SEM (*n* = 3) from three independent experiments. # *p* < 0.05 vs. control group (no treatment group); ** *p* < 0.01 and *** *p* < 0.001 vs. TGF-β1-stimulated group.

Moreover, we investigated the Smad-2/3-independent signaling pathways as downstream mechanisms for TGF-β1 signaling. TGF-β1 significantly augmented the phosphorylation of MAPKs and phosphoinositide 3-kinase/Protein kinase B (PI3K/Akt). As shown in Figure 4, SHE significantly suppressed ERK phosphorylation. SHE did not show any suppressive effect on the phosphorylation of JNK, p38 MAPK, and PI3K/Akt.

### 3.5. SHE Inhibits TGF-β1-Induced Nuclear Factor Kappa B (NF-κB) Activation in NPDFs

**Figure 5:**
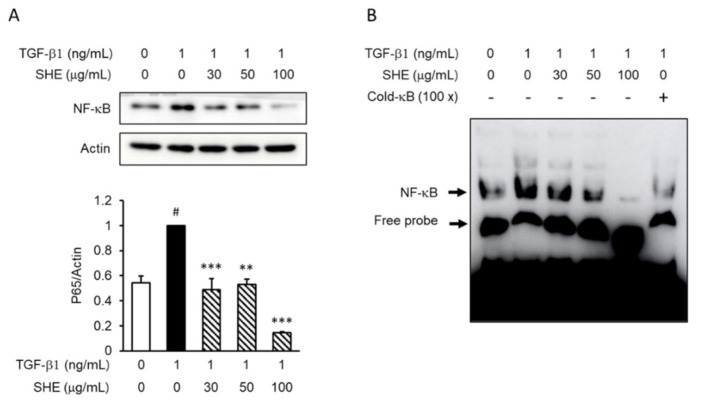
Effect of SHE on the activation of nuclear factor kappa B (NF-κB) in TGF-β1-induced NPDFs. The NPDFs were treated with SHE (30, 50, and 100 µg/mL) for 1 h and then stimulated with TGF-β1 for 1 h. (**A**) The level of nuclear translocation of NF-κB was analyzed using western blot with nuclei protein; (**B**) The binding activity of NF-κB was evaluated using electrophoretic mobility shift assay (EMSA). Each bar represents the mean ± SEM (*n* = 3) from three independent experiments. # *p* < 0.05 vs. control group (no treatment group); ** *p* < 0.01 and *** *p* < 0.001 vs. TGF-β1-stimulated group. Cold-κB, unlabeled NF-κB probe.

To elucidate the downstream mechanisms of the ERK pathway and nuclear transcription factors, we investigated the effects of SHE on the activation of NF-κB (Figure 5) in TGF-β1-stimulated NPDFs. Stimulation with TGF-β1 increased nuclear translocation and facilitated the DNA binding of NF-κB in the nuclei, whereas pretreatment with SHE markedly attenuated nuclear translocation (Figure 5A) and DNA binding of NF-κB (Figure 5B).

### 3.6. SHE Inhibits TGF-β1-Stimulated Fibroblast Contractile Activity

**Figure 6:**
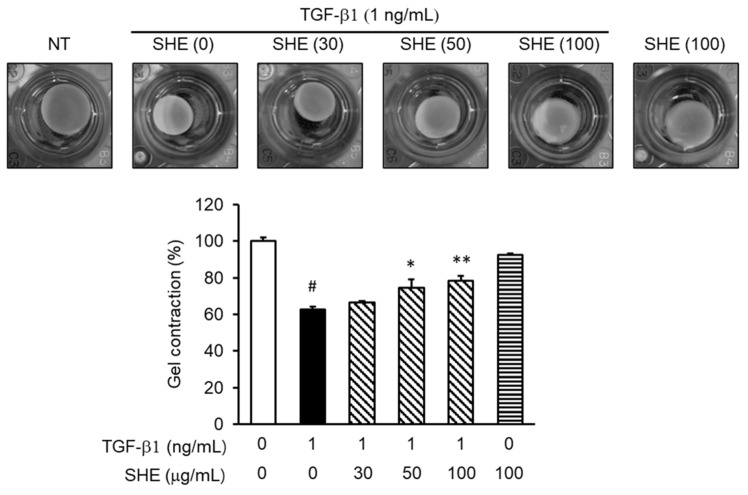
Effect of SHE on the contractile activity of NPDFs in rat tail type I collagen gel. Cells were applied in rat tail type I collagen gels (1 mg/mL) and pretreated with SHE for 1 h. Next, the mixture of collagen gel and the cell was treated with TGF-β1 (1 ng/mL) and then cultured for another 24 h. TGF-β1 stimulation decreased the gel size, while SHE pretreatment blocked this reduction in gel size. It was evaluated and quantified as the sum of the percentage of gel surface area in each well relative to the control gel surface area. Each bar represents the mean ± SEM (*n* = 3) from three independent experiments. # *p* < 0.05 vs. control group (no treatment group); * *p* < 0.05 and ** *p* < 0.01 vs. TGF-β1-stimulated group.

NPDFs were applied to a type I collagen gel, as described in the Section 2. The collagen gel mixture containing cells was then pretreated with SHE (30, 50, and 100 μg/mL) for 30 min, followed by TGF-β1 (1 ng/mL) stimulation for 24 h. The TGF-β1 stimulation led to a decrease in the collagen gel pad size (62.71% vs. TGF-β1-untreated group [100%]; # *p* < 0.05), but SHE pretreatment blocked gel contraction (30, 50, and 100 μg/mL SHE: 106.1%, 119.1%, 125.1% vs. TGF-β1-treated group (100%), respectively; * *p* < 0.05 and ** *p* < 0.01) depending on the concentration (Figure 6).

### 3.7. SHE Inhibits α-SMA, Collagen-1, and Fibronectin Protein Expression in Nasal Polyps Organ Culture

**Figure 7:**
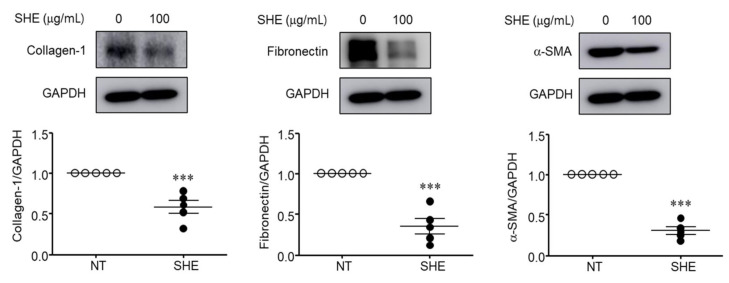
Effect of SHE on collagen-1, fibronectin, and α-SMA protein expression in NP tissues. The NP tissues were incubated with 100 μg/mL of SHE using an air–liquid interface organ culture method. Following incubation with SHE for 24 h, collagen-1, fibronectin, and α-SMA protein expression level was assessed by western blotting. Each value indicates the mean ± SEM (*n* = 5) and is representative of results obtained from three independent experiments. *** *p* < 0.001 vs. NT (no treatment group).

We performed organ cultures to investigate the antifibrotic effects of SHE on NP formation. We measured collagen-1, fibronectin, and α-SMA expression levels after applying 100 μg/mL SHE for 24 h using western blotting (Figure 7). The expression levels of collagen-1, fibronectin, and α-SMA were attenuated in SHE-treated NP tissues compared to those in non-treated NP tissues.

## 4. Discussion

*S. horneri* exhibits a broad range of biological activities. Therefore, we assessed the effects of *S. horneri* on fibrosis and its regulatory role in signaling pathways in NPDFs. NPs are associated with stromal fibrosis in the sinus, causing them to grow like pseudocysts. Thus, the suppression of fibrotic progression is thought to be the expected therapeutic management for NP recurrence after endoscopic sinus surgery for NPs. For this reason, we investigated the antifibrogenic effect of SHE and its effects on regulatory signaling in vitro in TGF-β1-induced NPDFs. In addition, we investigated the attenuation of deposition of collagen-1, fibronectin, and α-SMA expression ex vivo using SHE in NP tissues to support our hypothesis that SHE could be a possible preventive agent for NP recurrence after endoscopic sinus surgery for NPs.

Fibroblasts are much denser in the stroma of NPs; accordingly, they are a suitable therapeutic target for treating NP formation. The high accumulation of ECM proteins (collagen-1 and fibronectin) and myofibroblast (α-SMA) differentiation could be correlated with the morbidity of NPs. Myofibroblasts synthesize large amounts of ECM proteins, mainly collagen and fibronectin. This study used TGF-β1, which is regarded as a significant fibrogenic cytokine, to stimulate NPDFs in an in vitro fibrosis model. TGF-β1, a multifunctional cytokine, plays a critical role in the development and progression of fibrotic responses and induces fibroblast activation, proliferation, and differentiation [28,29]. Excessive elevation of TGF-β1, a key profibrotic driver, is involved in diverse fibrotic diseases, such as cirrhosis, Crohn’s disease, glomerulosclerosis, cardiac fibrosis, diabetic nephropathy, pulmonary fibrosis, and myocarditis in humans [28,30]. In an in vivo study, TGF-β1 induced the accumulation of ECM proteins, particularly collagen and fibronectin [31]. In addition, the enhanced α-SMA expression was decreased by inhibiting TGF-β1 expression. TGF-β1 was highly expressed in NP and in our preliminary findings (a previous study reported that TGF-β is expressed in nasal polyps [32]). These studies showed that increased TGF-β1 levels in NPs are closely involved in the pathogenesis of NP formation. Therefore, if the mechanisms induced by TGF-β1 are blocked during NP formation, it will be an effective preventive measure to suppress recurrent NP formation after surgical NP removal. Previous studies on NPDFs have shown that stimulation with TGF-β1 leads to ECM expression and myofibroblast differentiation. Based on these reports, we investigated the inhibitory effects of SHE on collagen-1, fibronectin, and α-SMA expression in TGF-β1-stimulated NPDFs. We found that SHE (30, 50, and 100 μg/mL) treatment significantly attenuated the expression of ECM proteins and differentiation of myofibroblasts in a concentration-dependent manner. Therefore, we expected that SHE would inhibit NP formation, especially the recurrence after endoscopic sinus surgery for NPs.

Next, we analyzed the TGF-β1-mediated critical signaling pathway to determine the mechanisms underlying the inhibitory effect of SHE on the fibrogenic response in NPDFs. TGF-β1 activates the canonical (Smad-dependent) and non-canonical (Smad-independent) pathways to exert multiple biological effects. In previous studies, the TGF-β1-mediated Smad-dependent signaling pathway was found to be significant in fibrosis [33]. When TGF-β1 binds to its receptors, such as type I and type II TGF-β receptors, it activates the Smad downstream signal. TGF-β1 binds to the receptor and induces the phosphorylation of Smad 2 and Smad 3. The activated Smad 2/3 forms oligomeric complexes with Smad 4, which is translocated to the nucleus. These complexes activate the transcription of profibrotic genes and consequently induce fibrotic responses [34]. We observed that SHE treatment attenuated the nuclear translocation of TGF-β1-induced phosphorylated-Smad 2/3 complexes (Figure 3A). To verify the role of Smad 2/3 in the regulatory effect of SHE on the expression of ECM proteins and differentiation of myofibroblasts, SIS3 was administered before TGF-β1 induction in NPDFs. The expression levels of collagen-1, fibronectin, and α-SMA were observed. As expected, SIS3 treatment significantly attenuated ECM protein expression and myofibroblast differentiation. These results demonstrated that SHE attenuated the fibrogenic response via the Smad mechanism in TGF-β1-stimulated NPDFs.

In addition to the Smad-dependent pathway, it also signals through other signal transducers for the TGF-β1-induced fibrotic response. In previous reports, TGF-β1-induced MAPK and PI3K/Akt activation was involved in the expression of collagen-1, fibronectin, and α-SMA in NPDFs [35]. We investigated the involvement of Smad-independent pathways in TGF-β1-induced signaling underlying the antifibrotic effects of SHE. As shown in Figure 4, SHE attenuated ERK activation, but did not inhibit TGF-β1-stimulated JNK, p38 MAPK, and PI3K/Akt phosphorylation. Furthermore, in a previous report, an ERK signal-specific inhibitor (U0126) blocked collagen-1, fibronectin, and α-SMA expression levels in TGF-β1-induced NPDFs [35]. This finding is consistent with the results of our study. Therefore, we suggest that ERK signaling is involved in the modulation of ECM protein production and myofibroblast differentiation.

To confirm the signaling mechanism more precisely, we investigated the downstream transcription factors of ERK, such as NF-κB. NF-κB expression has been reported to be augmented in CRSwNPs patients [36]. A previous study reported that NF-κB plays a crucial role in the expression of collagen-1, fibronectin, and α-SMA in TGF-β1-stimulated NPDFs [29]. Therefore, we investigated whether SHE (30, 50, and 100 μg/mL) modulates NF-κB activation. This study showed that SHE attenuated NF-κB activation in response to TGF-β1 stimulation, using western blotting and electrophoretic mobility shift assay (EMSA). Therefore, NF-κB plays a regulatory role in the expression of collagen-1, fibronectin, and α-SMA in SHEs in TGF-β1-stimulated NPDFs. Taken together, we propose that SHE suppresses TGF-β1-stimulated ECM production and myofibroblast differentiation via at least two different signaling pathways: Smad 2/3-dependent and -independent signaling pathways.

TGF-β1-induced gel contraction of fibroblast-populated collagen pads increases the mechanical load owing to elevated α-SMA expression levels [37]. The culture of fibroblasts in a three-dimensional collagen gel pad made of type I collagen has been used as a model for wound repair and fibrosis [38]. Therefore, we evaluated the effect of SHE on type 1 collagen gel pad contraction mediated by TGF-β1-stimulation. While stimulation with TGF-β1 decreased the fibroblast-populated collagen pads, treatment with SHE on the collagen gel pad inhibited contraction (Figure 6). This experiment suggests that SHE could inhibit TGF-β1-mediated tissue remodeling.

Finally, we explored the effects of SHE on the expression levels of collagen-1, fibronectin, and α-SMA in the NP organ culture model. SHE significantly inhibited the expression of collagen-1, fibronectin, and α-SMA (Figure 7). These results suggest that SHE can be used as a therapeutic agent for NP recurrence after endoscopic sinus surgery for NP.

In conclusion, our results demonstrate that SHE effectively suppressed TGF-β1-induced ECM protein expression and myofibroblast differentiation by blocking the activation of Smad 2/3 and ERK/NF-κB signaling pathways in NPDFs. In addition, SHE significantly inhibited the contraction of the collagen gel pad. Furthermore, in an NP organ culture model, SHE showed a distinct suppressive effect on ECM protein accumulation and myofibroblast differentiation. Our findings suggest that SHE may be a potential therapeutic agent for treating NP recurrence after endoscopic sinus surgery for NP. Besides, the major peak in SHE was identified as Apo-9-fucoxanthinone by LC-DAD-ESI/MS (Figure S1). The previous study has suggested that Apo-9-fucoxanthinone possesses potent anti-inflammatory and antioxidant activity [16]. Therefore, it was hypothesized that the antifibrotic effect of SHE is derived from Apo-9-fucoxanthinone. To further confirm whether the antifibrotic effect of SHE is due to the presence of Apo-9-fucoxanthinone should be explored in future studies.

## Data Availability

All data generated during this study are included in this published article.

## Supplementary Materials

The following supporting information can be downloaded at: https://www.mdpi.com/article/10.3390/cimb44110395/s1, Figure S1: Mass spectra of the Apo-9 fucoxanthinone isolated from *Sargassum horneri*. Reference [39] is cited in Supplementary Material.
