# Supplementary material for: Antifibrosis Efficacy of Apo-9-Fucoxanthinone-Contained Sargassum horneri Ethanol Extract on Nasal Polyp: An In Vitro and Ex Vivo Organ Culture Assay
Source: Curr Issues Mol Biol. 2022 Nov 21;44(11):5815–26. doi: 10.3390/cimb44110395 (PMC9689088; doi:10.3390/cimb44110395)
Supplement: Supplementary file 1 [file cimb-44-00395-s001.zip › cimb-1994892-supplementary.pdf]

Article

Antifibrosis efficacy of Apo-9-fucoxanthinone-contained *Sargassum horneri* ethanol extract on nasal polyp: an *in vitro* and *ex vivo* organ culture assay

Mi-Jin Yim [1], Jeong Min Lee [1], Seok-Chun Ko [1], Hyun-Soo Kim [1], Ji-Yul Kim [1], Seong Kook Park [2], [1] Dae-Sung Lee [1,*] and Il-Whan Choi [3,*]

1 National Marine Biodiversity Institute of Korea, Seocheon, Chungcheongnam-do 33662, Republic of Korea
2 Department of Otorhinolaryngology-Head & Neck Surgery, Inje University College of Medicine, Busan Paik Hospital, Busan 47392, Republic of Korea
3 Department of Microbiology and Immunology, College of Medicine Inje University, Busan 47392, Republic of Korea
* Correspondence: cihima@inje.ac.kr; daesung@mabik.re.kr

List of Figure

Figure S1. Mass spectra of the Apo-9 fucoxanthinone isolated from *Sargassum horneri*.

HPLC-DAD-ESI/MS analysis of Apo-9-fcoxanthinone

HPLC-DAD-ESI/MS was progressed using the assay described by Kim el al. [Reference 1]. The analysis was carried out using an HPLC-DAD system (Hewlett-Packard, Waldbronn, Germany) coupled with a Finnigan MAT LAW ion-trap mass spectrometer (ESI/MS, Finnigan MAT, San Jose, CA, USA). The positive ion mass spectra were recorded in the range m/z 100-2000. The source voltage was set to 5 kV while the capillary temperature was 275 ℃. The capillary voltage was maintained at 36.5 V; inter-octapole lens voltage was at 10 V; sheath gas 80 psi (551.6 kPa); and auxiliary gas, 20 psi (137.9 kPa).

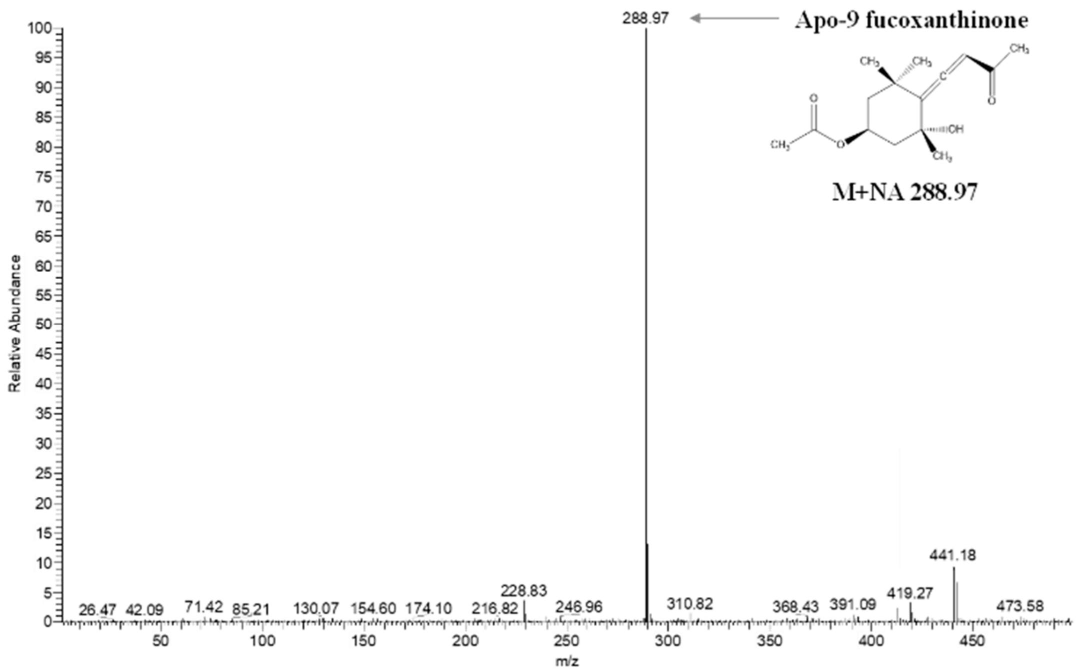

Figure S1. Mass spectra of the Apo-9 fucoxanthinone isolated from *Sargassum horneri*. The major peak in *Sargassum horneri* extract was determined to Apo-9-fucoxanthinone by LC-DAD-ESI/MS. The molecular weight of Apo-9-fucoxanthinone is identified as 265 Da.

Reference

1. Kim, H.-S.; Wang, L.; Jayawardena, T.U.; Kim, E.A.; Heo, S.J.; Shanura, I.P.S.; Lee, J.H.; Jeon, Y.J. High-performance centrifugal partition chromatography (HPCPC) for efficient isolation of diphlorethohydroxycarmalol (DPHC) and screening of its antioxidant activity in a zebrafish model. *Process Biochem*. **2020**, 88, 189–196. doi: 10.1016/j.procbio.2019.09.027.
